# President’s Address1Delivered before the Veterinary Medical Association of New Jersey, at Trenton, January 8, 1903.

**Published:** 1903-02

**Authors:** William Herbert Lowe

**Affiliations:** Paterson, N. J.


### PRESIDENT'S ADDRESS.[1]

BY WILLIAM HERBERT LOWE, D.V.S.,
PATERSON, N. J.

FELLOW-MEMBERS AND COLLEAGUES: The veterinarian is a student of life. The Bible and scientific research agree that vegetable and animal life existed on this earth before man, and the veterinarian believes in beginning his studies at the beginning for the benefit of animal life in general as well as for the benefit of man himself. There are two worlds of life that man cannot see unaided. In one the organisms are so infinitesimally small that they cannot be seen with the naked eye, and in the other the beings cannot be seen with the naked eye because they are beyond his horizon. As man has dominion over the lower animals it is incumbent upon him, in the very nature of things, that certain men should make a study and

[1] Delivered before the Veterinary Medical Association of New Jersey, at Trenton, January 8, 1903.

practice of animal or veterinary science for the benefit of our material and business prosperity, as well as for such knowledge as concerns the health and lives of ourselves.

Some people do not like the profession of the veterinarian, because in his practice he has to go into stables, treat the accidents and ailments of animals, and come in contact with attendants of animals.

I would like to remind these people of the incident of Christ's birth; how He was born in a stable, wrapped in swaddling clothes, and laid in a manger with the cattle. He was not contaminated, and neither will anyone of to-day be if he is properly constituted and has right and noble impulses. I allude to the incident of Christ's birth at this time simply to remind superficial people that it is always well to have a full understanding of any subject before passing judgment upon it.

Experiments made upon animals during the last few years, the study of vegetable and animal life in its elementary and minutest forms, together with recent discoveries in chemistry, are exceedingly rich in the results of new discoveries and important investigations which have revolutionized the previously conceived ideas of medical men as to the etiology of disease, which are not only of great practical importance to the veterinarian in his practice, but are of vital importance to mankind, since they furnish the foundation for modern and intelligent treatment of many of the diseases that afflict the human family.

The fundamental facts of the pathology of the more common of the infectious diseases of animals in the light of our present day knowledge will be given us to-day in a concise form, and illustrated by the stereopticon by one who stands in the front rank among American comparative pathologists and bacteriologists.

During the last three years the veterinary profession of New Jersey has been making history that will ever be of increasing benefit and advantage to the people of our State, and an enduring honor to the veterinarians who made possible the triumph and achievements that we record to-day. I will not dwell upon the recent struggles and labors of many loyal members of this organization, for the work they did and the personal sacrifices they made are still fresh in our memories.

Veterinary annals will record three triumphant days in New Jersey. The first is January 11, 1900, which day witnessed one of the most remarkable gatherings that ever occurred in the history of veterinary medicine, when there gathered together the largest number of veterinarians that had ever assembled at one time in New Jersey, and resulted in the surrender of two State charters and seals and the successful and complete amalgamation of three State societies in one strong, harmonious body.

The second triumphant day was March 17, 1902, when Governor Franklin Murphy signed Senate Bill No. 76, by Senator Wood McKee, entitled "An act to regulate the practice of veterinary medicine, surgery, and dentistry in the State of New Jersey, to license veterinarians, and to punish persons violating the provisions thereof," thus making this bill a sovereign law of our State. This law is known as Chapter 18, Laws of 1902.

The third triumphant day was May 5, 1902, when the five members of the State Board of Veterinary Medical Examiners appointed by Governor Murphy took the oath of office in the State House and organized the veterinary board, pursuant to the provisions of the new law.

I wish to emphasize the fact that the enactment of this law, creating a State board of veterinary medical examiners to regulate the practice of veterinary medicine, surgery, and dentistry in the State of New Jersey, to license veterinarians and to punish persons violating the provisions thereof, was made in recognition of the necessity and value of competent veterinary service to live-stock owners, agricultural and dairy interests, and the preservation of public health; in other words, to protect the public from charlatans and quacks rather than to protect competent veterinarians, for the qualified veterinarian does not need any such protection.

The movement inaugurated this year to organize local societies in the respective counties of the State in affiliation with the Veterinary Medical Association of New Jersey, is one to which I attach a great deal of importance. I am very proud to say that the movement was started in my own county, and that on July 7, 1902, the practitioners of this county organized the Passaic County Veterinary Medical Association, which meets monthly, and the meetings are well attended. Every licensed practitioner in the county, now nineteen in number, is an earnest member and staunch supporter of the local organization. Every member signed the constitution, by-laws, code of ethics, and schedule of fees adopted by the society. The members are all pledged, *upon their honor*, to support the constitution, by-laws, code of ethics, and schedule of fees. I am gratified and delighted to be able to say that each member takes much pride in fulfilling his obligations and extending courtesies to his brother practitioners, that add much to the pleasure and satisfaction of practice. Local societies should be organized in such counties as have a sufficient number of practitioners to warrant it, each in affiliation with the State association.

One of the most noteworthy events in the veterinary world that occurred during the first year of the twentieth century was the meeting of the American Veterinary Medical Association at Atlantic City, during the first week in September, 1901, where gathered together the representative veterinarians of the entire American continent. It was the distinguished privilege of the Veterinary Medical Association of New Jersey to welcome and entertain the veterinary hosts at this Mecca by the sea. The achievements of this meeting add also to the recent history the profession has been making in New Jersey.

The State Laboratory of Hygiene is now located at Trenton, under the supervision of the State Board of Health. The work is conducted free of charge and it consists in examination for diagnosis in the various affections which are produced by micro-organisms. Communicable diseases of whatever character, whether peculiar to man or to the lower animals, are investigated, and a diagnosis is made when possible. Veterinarians should avail themselves of the use of the laboratory in cases of communicable diseases. The State Board of Health furnishes blanks for reporting contagious diseases in animals. Veterinary practitioners should report all cases of contagious diseases to the State Board of Health on the blanks furnished by the Board.

In the year 1862, an act was passed by Congress providing for the establishment of State agricultural colleges for the teaching of agriculture and mechanical arts; and in 1887 Congress passed an act establishing agricultural experiment stations in connection with agricultural colleges for original research into "the physiology of plants and animals, the disease to which they are severally subject, with the remedies for the same."

In other words, Congress several years ago provided for original research in veterinary science as well as for its practical application. I have not had an opportunity, much as I should have liked to have had, of visiting our State Agricultural College and Experiment Station. I would recommend that this Association appoint a committee to make such visit and ascertain what is being done along the lines of modern veterinary science and art, and if this branch of the work is in the hands of qualified veterinarians, or whether laymen and men of other professions are engaged in it.

I would recommend that this Association adopt a two days'

session at its annual meetings, and that it start a State Veterinary Library. Your President's office has been virtually a veterinary bureau of information during the last two years. The correspondence has been such as to require much time and daily attendance.

Among the positions that New Jersey veterinarians have filled with credit during the past two years are: Health Officer of Summit; Assistant Bacteriologist of the Health Department of Newark; Veterinarian to the Essex Troop; Veterinarian to the Atlantic City Horse Show; Chairman and Members of Committee on Animal Diseases and Animal Food of the New Jersey Sanitary Association; Veterinarian to Local and City Boards of Health, and City Veterinarian to some of our larger cities.

I wish in behalf of the Veterinary Medical Association of New Jersey to take this opportunity to publicly acknowledge the great debt of gratitude the profession of our State owes to the *American Veterinary Review* and to the JOURNAL OF COMPARATIVE MEDICINE AND VETERINARY ARCHIVES. One of the greatest sources of encouragement in the work of organization and legislation was the ever earnest and able support by both these journals. Veterinary journalism in this country has become a power in the profession. I do not believe that there is another single factor that is equal to the veterinary press in promoting the individual and common interests of the profession.

The veterinarian cannot be up to date in his knowledge and his methods of practice, unless he knows what is going on in the veterinary world. I cannot see how a veterinarian can get along without his *Review* or his JOURNAL. If he does, he is simply starving his mind of such information as would go to enrich it and make him a better and more successful practitioner. He is not only doing an injustice to himself by not keeping in touch with the advance work of the profession, but he is doing an injustice to his clients, and as a natural consequence he is the sufferer in the end. There is no investment that pays a practitioner such large returns as his subscription to the profession's periodicals and his dues to his State Association.

Do not neglect to contribute your share of knowledge and experience to the columns of the veterinary press. I would suggest that you do not confine your reports to those of cases of exceptional and rare occurrence, for it is often in some little point in connection with routine manipulation, or in the treatment of the more common diseases, that the greatest benefit is derived from reporting and recording your experience.

A recent poll of one of the most prosperous of the Western States developed the amazing fact that there were nearly one hundred graduated veterinarians in that State who are not subscribers to any American or foreign veterinary journal. Any comment is unnecessary. There are two things, I am sure, that every progressive, up-to-date veterinary practitioner of New Jersey will not be found wanting in—one is, he will be found to be a staunch and earnest member of the Veterinary Medical Association of New Jersey, and the other is, that he is a subscriber to at least one veterinary periodical.

It seems to me that it would be a great advantage in many ways if we had a State board or bureau of animal industry, with a State Veterinarian who would be Chief of the said board or bureau, somewhat on the same plan as the United States Bureau of Animal Industry, instead of having veterinary matters of the State connected with several of the different State bodies without professional directorship, as is now the case. It might be well for this Association to appoint a conference committee to see if a plan could not be adopted that would be satisfactory to the State Board of Agriculture, the State Board of Health, the Tuberculosis Commission, and all concerned. It is all right to have veterinarians make inspections and so on, but there should be a State Veterinarian at Trenton to direct the work along modern scientific lines. As soon as plans can be perfected that will be satisfactory to the various bodies concerned, I would recommend that steps be taken to secure the necessary legislation. Our agricultural and dairy interests, as well as the preservation of the public health, demand that a qualified veterinarian be the chief or director of the veterinary work of the State.

Gentlemen, just think of it! The Legislature last winter authorized our good friend the State Entomologist to chase mosquitoes, and appropriated $10,000 for his use (which was proper), yet the animal wealth of this entire State, including our great dairy industry, supplying much of the milk for the cities of New York and Philadelphia, is left without the supervision of a qualified State veterinarian at Trenton.

I cannot refrain, before concluding this address, from making a few personal remarks. The loyal and enthusiastic support that every officer and member of this amalgamated association has given me as your executive officer during the last two years has been such as to strengthen my hands, without which the achievements we recount to-day would have been nothing but poor, miserable failures.

This gavel, the silver service, as well as the resolutions you passed on the loss I sustained in the terrible conflagration on the night of February 9th last, when my offices, veterinary buildings and appointments, pharmacy, library, instruments, and many things that money cannot replace, were destroyed, were kind tokens from you that bespeak more than language can express.

You may be interested in knowing that the original draft of our veterinary law was destroyed in that fire. It was in Senator McKee's office, where he and I had gone over it together the day previous. This must be a good law, for it can truthfully be said that neither fire nor flood could impair or kill it.

Aside from general practice, I see two large, uncultivated, and sadly-neglected fields for the qualified veterinarian. One is that of animal husbandry and industry; the other is that of veterinary sanitary medicine and police. The public is beginning to demand that adequate safeguards be placed around their meat and milk-supply, and I am sure that this Association in its wisdom will not shirk its duty, but on the contrary place the veterinary profession in a position to deal successfully with the important issues that are sure to confront it. Let every veterinarian continue to do his part, be it little or much, and the time is not far distant when the profession will have the satisfaction of having one of its own members as the director of veterinary affairs at Trenton, and the public the benefit of a veterinary service operated upon the basis of modern scientific knowledge and experience, that will add in a large degree, and in a real and substantial manner, to the health and wealth of the people of our beloved State.

